# Optimising infection prevention and control practice using behavior change: a systematic review

**DOI:** 10.1186/1753-6561-5-S6-P274

**Published:** 2011-06-29

**Authors:** R Edwards, E Charani, N Sevdalis, B Alexandrou, E Sibley, D Mullett, HP Loveday, LN Drumright, A Holmes

**Affiliations:** 1 The National Centre for Infection Prevention and Management, London, UK; 2 Department of Surgery and Cancer and Imperial Centre for Patient Safety and Service Quality, Imperial College London, London, UK; 3 Dr Foster Intelligence, London, UK; 4 Faculty of Health & Human Sciences , University of West London, London, UK; 5 Imperial College Healthcare NHS Trust, London, UK

## Introduction / objectives

Despite significant investment in infection prevention and control (IPC), there has been little consideration of the effectiveness of behaviour change interventions or the application of behavioural theory (BT) or social marketing (SM) to influence healthcare workers’ (HCWs) behaviour and to reduce healthcare associated infection.

## Methods

This review used a systematic process to assess the effectiveness of intervention studies focused on behaviour change within IPC and the extent to which BT or SM has been applied. Exploratory studies that seek to understand influences on HCWs behaviour in IPC were reviewed. We searched MEDLINE, EMBASE, ASSIA, Business Source Complete, The Cochrane Library, PsycINFO, DARE and HMIC for studies performed between 1999-2009.

## Results

Twenty studies met the quality criteria. Few behaviour change interventions of sufficient methodological quality or adequate evaluation design were identified. Of the seven included intervention studies, few explicitly considered BT, used SM or addressed sustainability. Exploratory studies highlighted some key social and cultural determinants of HCW behaviour in IPC.

## Conclusion

The quality of interventions aimed at changing the IPC behaviours of HCWs is poor and there has been limited application of BT or SM in methodologically robust study designs. We recommend that quality criteria be established and added to existing guidelines to ensure robust design, implementation and reporting of behaviour change interventions in IPC.

## Disclosure of interest

None declared.

